# Carotenoid dynamics and lipid droplet containing astaxanthin in response to light in the green alga *Haematococcus pluvialis*

**DOI:** 10.1038/s41598-018-23854-w

**Published:** 2018-04-04

**Authors:** Shuhei Ota, Aya Morita, Shinsuke Ohnuki, Aiko Hirata, Satoko Sekida, Kazuo Okuda, Yoshikazu Ohya, Shigeyuki Kawano

**Affiliations:** 1 Department of Integrated Biosciences, Graduate School of Frontier Sciences, The University of Tokyo, Kashiwanoha, Kashiwa, Chiba, 277-8562 Japan; 2 Bioimaging Center, Graduate School of Frontier Science, The University of Tokyo, Kashiwanoha, Kashiwa, Chiba, 277-8562 Japan; 3 Graduate School of Kuroshio Science, Kochi University, Akebono-cho, Kochi, 780-8520 Japan; 4 Present Address: Center for Environmental Biology and Ecosystem Studies, National Institute for Environmental Studies, Tsukuba, Ibaraki, 305-8506 Japan; 5 Present Address: Future Center Initiative, The University of Tokyo, Wakashiba, Kashiwa, Chiba, 277-0871 Japan

## Abstract

The unicellular green alga *Haematococcus pluvialis* accumulates large amounts of the red ketocarotenoid astaxanthin to protect against environmental stresses. *Haematococcus* cells that accumulate astaxanthin in the central part (green-red cyst cells) respond rapidly to intense light by distributing astaxanthin diffusively to the peripheral part of the cell within 10 min after irradiation. This response is reversible: when astaxanthin-diffused cells were placed in the dark, astaxanthin was redistributed to the center of the cell. Although *Haematococcus* possesses several pigments other that astaxanthin, the subcellular distribution and content of each pigment remain unknown. Here, we analyzed the subcellular dynamics and localization of major pigments such as astaxanthin, β-carotene, lutein, and chlorophylls under light irradiation using time-lapse and label-free hyperspectral imaging analysis. Fluorescence microscopy and freeze-fracture transmission electron microscopy showed that, preceding/following exposure to light, astaxanthin colocalized with lipid droplets, which moved from the center to the periphery through pathways in a chloroplast. This study revealed that photoresponse dynamics differed between astaxanthin and other pigments (chlorophylls, lutein, and β-carotene), and that only astaxanthin freely migrates from the center to the periphery of the cell through a large, spherical, cytoplasm-encapsulating chloroplast as a lipid droplet. We consider this to be the *Haematococcus* light-protection mechanism.

## Introduction

Land plants and algae have evolved strategies for adapting to changes in environmental conditions, such as sunlight intensity. Chloroplast photorelocation is a crucial physiological response to high-intensity light. The signaling components of organelle photorelocation have been investigated in the model plant *Arabidopsis*^1–4^. Unlike terrestrial plants, *Haematococcus* cells possess one large, cup-shaped immovable chloroplast^5^. *Haematococcus* cysts, which are sessile cells lacking flagella, have adapted to respond to intense light by accumulating carotenoids. The cup shape closes to become a large, spherical chloroplast-encapsulating cytoplasm during encystment^6^.

The unicellular green alga *Haematococcus pluvialis* accumulates large amounts of the red ketocarotenoid astaxanthin, which plays an important role in protecting plants and algae from environmental stressors^7–11^. Under stress, such as intense light, *Haematococcus* cells increase the synthesis and accumulation of astaxanthin^12,13^. Astaxanthin is synthesized from β-carotene as a precursor in a reaction catalyzed by β-carotene ketolase. Chen *et al*. proposed a model of astaxanthin biosynthesis in *Haematococcus*; β-carotene is exported from the chloroplast to the endoplasmic reticulum (ER), where it is converted into astaxanthin, then astaxanthin esters, and finally forms astaxanthin ester-containing lipid droplets^13^. This enzyme plays an essential role in the stress-dependent initiation of astaxanthin synthesis^12^.

*Haematococcus* astaxanthin is deposited in extra-plastidial oil globules (lipid droplets)^14^. *Haematococcus* astaxanthin is considered a high value-added substance, e.g., as a source of pigment in fish aquaculture and cosmetics, because of its antioxidative activity and red coloration^15–20^. Although *Haematococcus* cells contain carotenoids other than astaxanthin^21^, astaxanthin is the only carotenoid that accumulates in oil globules, where triacylglycerol is the major lipid^22^.

Recent bioimaging technology has enabled estimates of the carotenoid distribution in microalgae *in vivo*. Confocal Raman spectroscopy facilitates the determination of the distribution and concentration of astaxanthin during *Haematococcus* red cyst cell formation *in situ* and *in vivo*^23^ and helps classify the nutrient status of microalgal cells^24^. Collins *et al*. spectrally resolved the resonance-enhanced Raman signatures associated with astaxanthin and β-carotene, as well as chlorophyll fluorescence^25^. Hyperspectral imaging allows the collection of spectroscopic light data from ultraviolet to near-infrared wavelengths^26^. In recent years, it has been applied to microscopic imaging in cell biology to estimate intracellular parameters *in vivo* and in real time^27–30^. A major advantage of hyperspectral imaging is its ability to correlate signals at multiple wavelengths due to its superior spatial and spectral resolution. Each hyperspectral image in a stack is a (*x*, *y*, *λ*) data cube containing spatial and spectral information^29,30^. Nogami *et al*. demonstrated the intracellular distribution of carotenoids and chlorophylls in *Haematococcus* at various life-cycle stages using hyperspectral imaging combined with the image-processing software HaematoCalMorph^31,32^.

Peled *et al*. reported that intermediate *Haematococcus* cells that accumulate astaxanthin in their centers (hereafter, “green-red cyst cells”) respond to intense light by rapidly redistributing astaxanthin throughout the cell^33^. They suggested two mechanisms for the migration of oil globules containing astaxanthin: (i) reorganization of the chloroplast and (ii) close alignment of oil globules with the ER membrane. Oil globules containing astaxanthin reportedly migrate from the center to the periphery of the cell in response to intense light. Moreover, an analysis of the effects of light color and inhibitors on the movement of astaxanthin suggested that actin is involved in astaxanthin migration under blue light irradiation. Finally, freeze-fracture (FF) scanning electron microscopy (SEM) indicated that oil globules pass through a passage formed in the chloroplast. However, the relationship between astaxanthin and other pigments (β-carotene, lutein, and chlorophyll *a* and *b*) in photoresponse has not been fully elucidated. In addition, the relationship between lipid droplets and astaxanthin also remains to be determined. Their dynamics can be investigated by observing green-red cysts.

In this study, we analyzed localization of astaxanthin, β-carotene, lutein, and chlorophyll *a* and *b* following light irradiation using a hyperspectral camera. Lipid droplets containing astaxanthin dynamics were observed to move freely from the center to the periphery of the cell through a large, spherical, cytoplasm-encapsulating chloroplast under light and fluorescence microscopy and rapid-freezing/FF-TEM. Possible mechanisms of astaxanthin globule migration in response to intense light are discussed.

## Results

### Live imaging of astaxanthin movement

**Figure 1:**
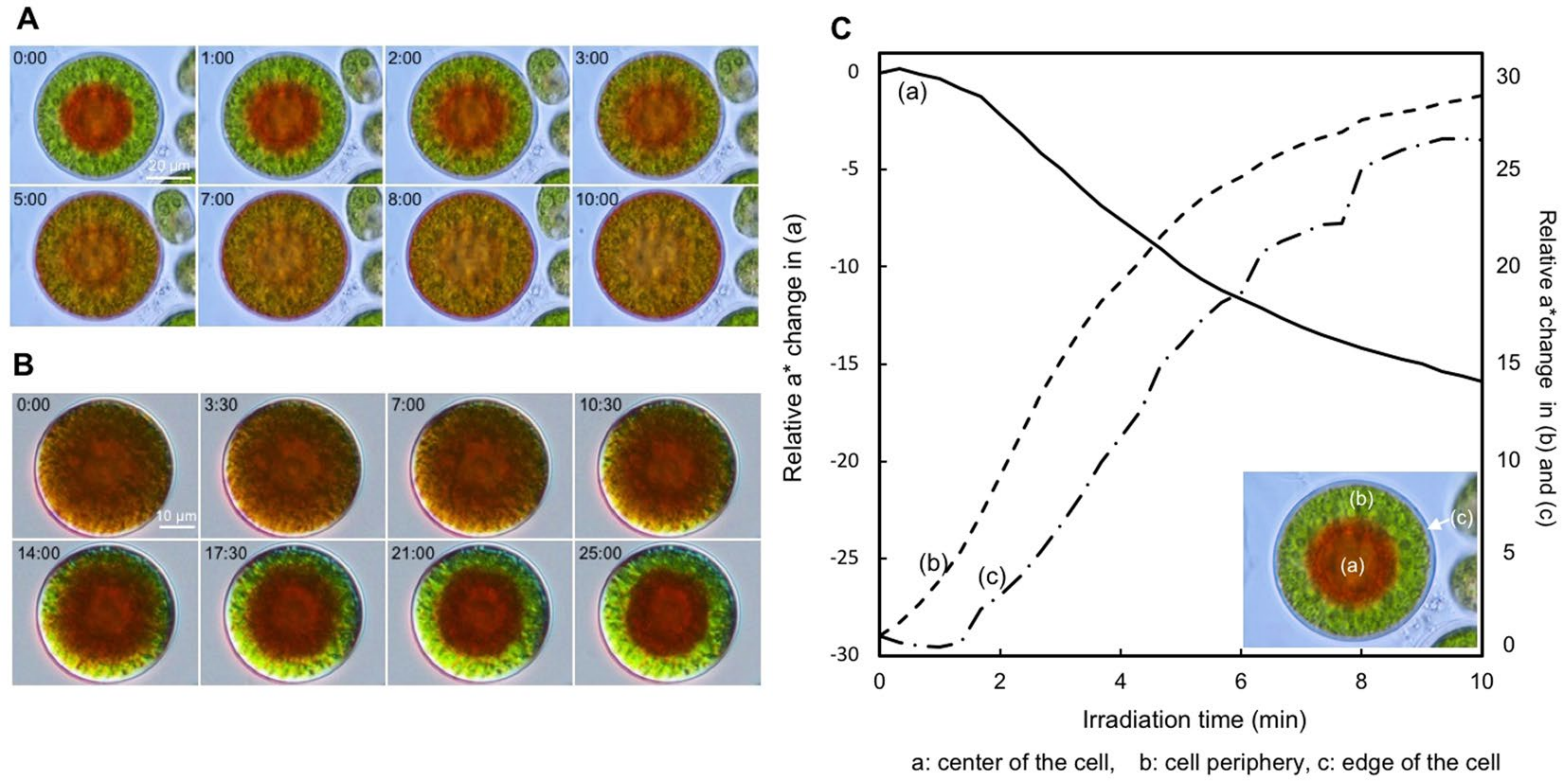
Time-lapse observation of astaxanthin diffusion and re-aggregation in green-red *Haematococcus* cells. (**A**) Time-lapse images at 0 to 10 min. Irradiation started at time 0 min. Time in minutes is shown at upper left. The panel is accompanied by Movie S1. (**B**) Time-lapse images at 0 to 25 min under dark conditions. Irradiation stopped at time 0 min. Time in minutes is shown at upper left. The panel is accompanied by Movie S2. (**C**) Temporal analysis of the red hue (a*) in three ROIs. Horizontal axis, time from the start of light irradiation; vertical axis, a* values. (**a–c**) are ROIs at the cell center, periphery, and beneath the cell wall, respectively (inset).

Astaxanthin in green-red cyst cells was localized in the central part of the cell (nuclear periphery) under normal light conditions (<45 µmol photons m^−2^ s^−1^) (Fig. 1A, time: 0 min). Time-lapse images obtained for 15 min at 15-s intervals showed that astaxanthin at the cell center rapidly diffused to the peripheral part of the cell within 10 min (Fig. 1A, Movie S1). In a cell in which astaxanthin was completely transferred to the periphery, a thin layer of astaxanthin was visible immediately beneath the cell wall (Fig. 1A, time 10 min). When astaxanthin-diffused cells were placed in the dark, astaxanthin was redistributed to the center of the cell (around the nucleus periphery) (Fig. 1B, Movie S2). However, a ring-shaped layer of astaxanthin remained near the cell wall (Fig. 1B, time 25 min). The kinetics of the movement of astaxanthin differed between the central and centrifugal directions (Fig. 1A,B). The former occurred within 10 min, but the latter required more than double that time to aggregate completely in the center of the cell.

**Figure 2:**
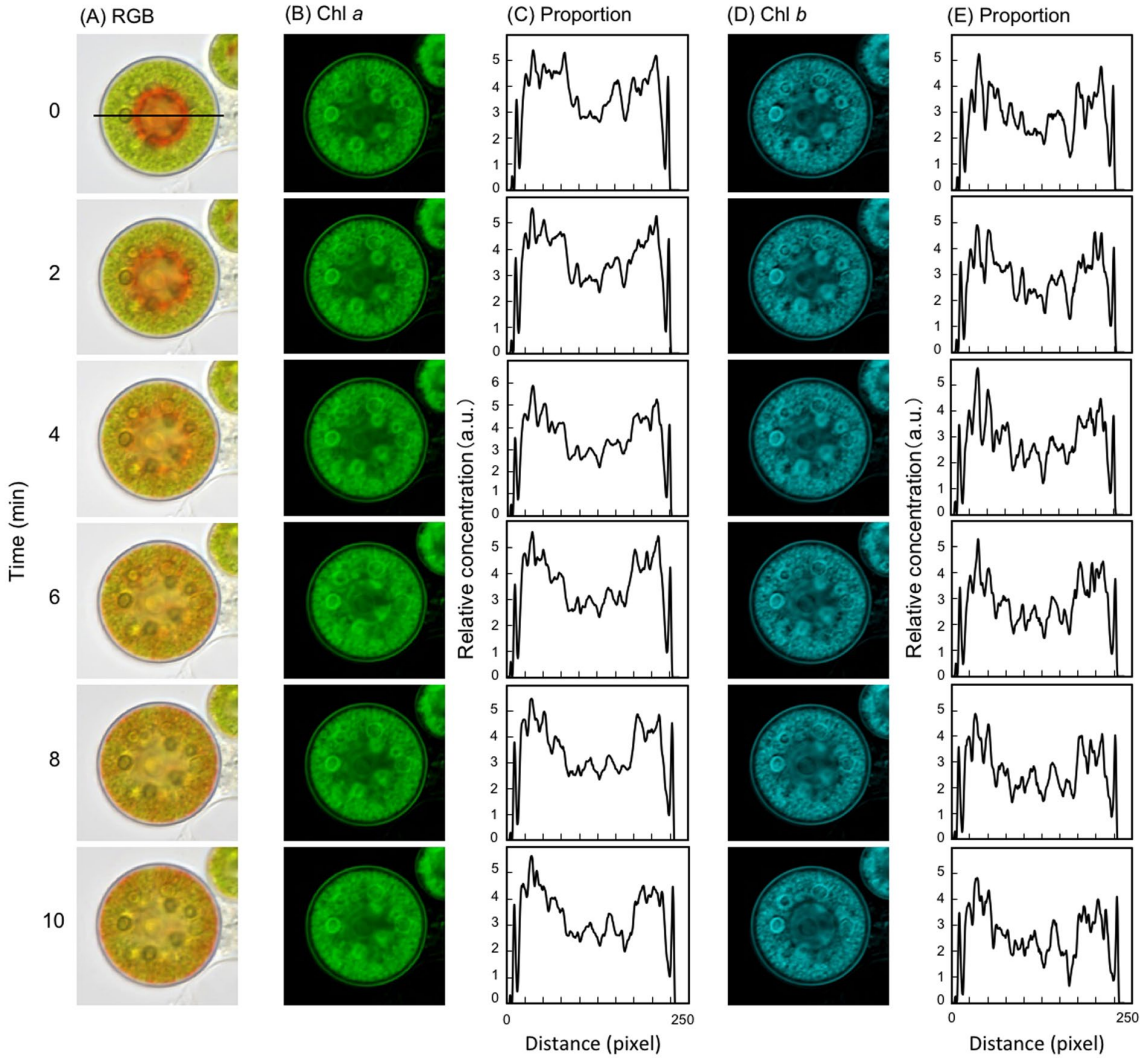
Intracellular chlorophyll dynamics revealed by time-course hyperspectral images. Images and graphs are arranged vertically at 2-min intervals. Irradiation started at time 0 min. The abscissa indicates the pixel distance of the central horizontal line of interest (middle region of the cell; e.g., (**A**) 0 min), and the ordinate indicates the relative amounts of each pigment. (**A**) RGB images of a green-red cyst. Irradiation started at time 0 min. (**B**) Hyperspectral pseudocolor images of the localization of chl *a*. (**C**) Distribution of the relative amount of the proportion of chl *a*. (**D**) Hyperspectral pseudocolor images of the localization of chl *b*. (**E**) Distribution of the relative amount of the proportion of chl *b*.

We used color-based segmentation and L*a*b* color space analysis to quantitatively evaluate time-lapse images of astaxanthin migration. The L*a*b a color-opponent space comprises the L* axis for lightness, the a* axis for red/green opponent colors [green at negative (−) a* values and red at positive (+) a* values], and the b* axis for yellow/blue opponent colors (blue at −b* values and yellow at +b* values). Because astaxanthin is a red pigment, the a* value was used here. After light irradiation (constant light using a 12 V/100 W microscope halogen lamp; see *Methods*), 41 time-lapse images of green-red cyst cells were taken at 15-s intervals using an inverted microscope, and the images were resolved by ImageJ software into the L*a*b* space. A region of interest (ROI) was selected in the central region of a green-red cyst cell, the chloroplast region, and immediately beneath the cell wall (Fig. 2C, inset); the red hue (a*) was then plotted (Fig. 2C). After 5 min, the a* value fluctuated markedly, and then stabilized from 8 to 10 min. The whole cell exhibited a red color after 5 min, and became uniformly red after 10 min (Fig. 1A,B).

### Hyperspectral imaging of carotenoid distribution *in vivo*

**Figure 3:**
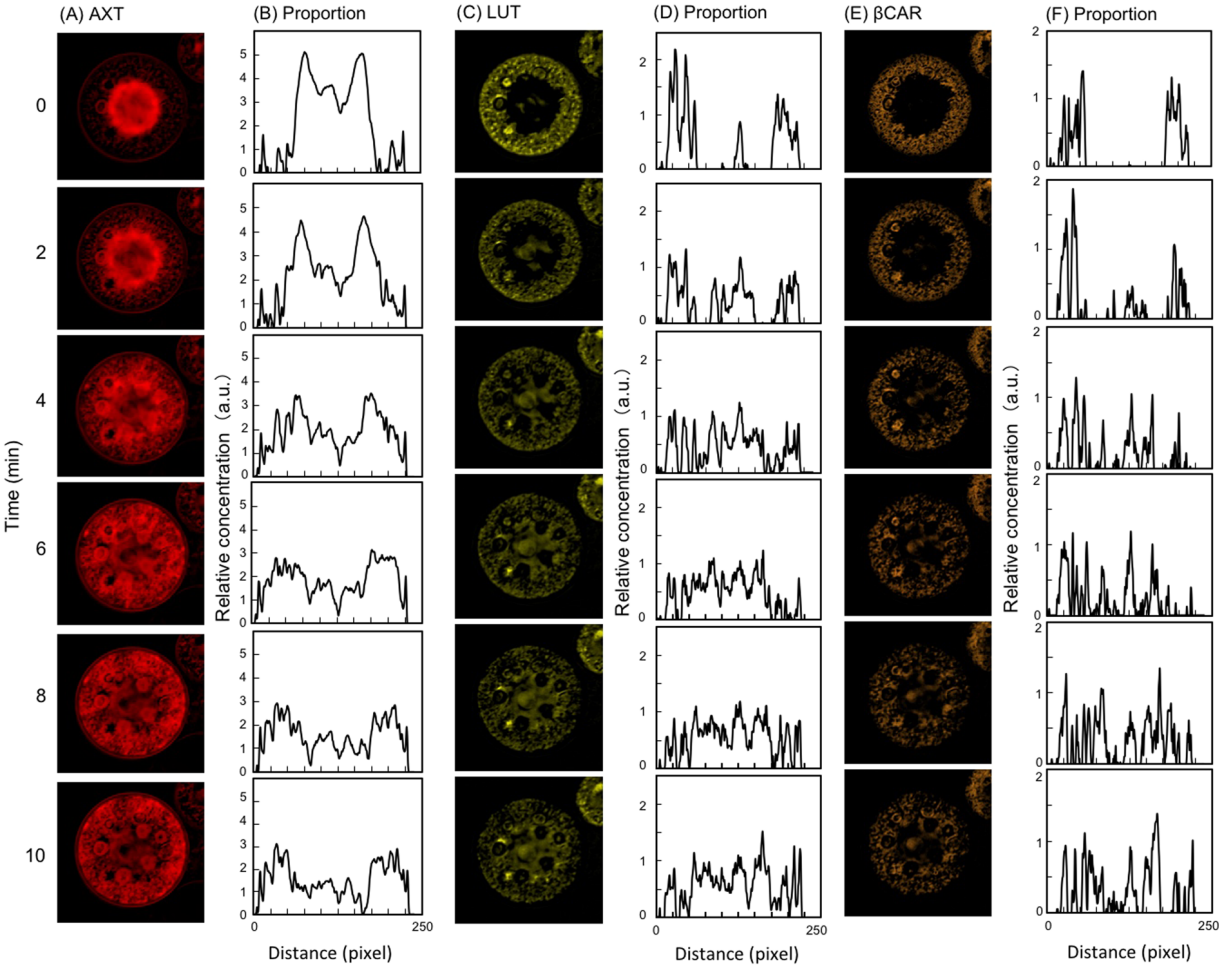
Intracellular carotenoid dynamics revealed by time-course hyperspectral images. Images and graphs are arranged vertically at 2-min intervals. Irradiation started at 0 min. The abscissa indicates the pixel distance of the central horizontal line of interest (middle region of the cell), and the ordinate indicates the relative amounts of each pigment. (**A**) Hyperspectral pseudocolor images of the localization of astaxanthin. (**B**) Distribution of the relative amount of astaxanthin. (**C**) Hyperspectral pseudocolor images of the localization of lutein. (**D**) Distribution of the relative amount of lutein. (**E**) Hyperspectral pseudocolor images of the localization of β-carotene. (**F**) Distribution of the relative amount of β-carotene.

The amount and distribution of intracellular pigment were estimated by hyperspectral imaging analysis, in which multiple wavelength spectral data can be obtained for each pixel. Nonnegative least square (nnls) analysis was applied to estimate the concentrations of astaxanthin, β-carotene, lutein, and chlorophyll *a* and *b*. Images were visualized in grayscale (pseudocolor) for each pigment, and grayscale values were converted into numerical data (2^16^ gray levels) using ImageJ. In addition, the horizontal axis of the ROI passing through the cell center was plotted as a pixel distance (pixel size, 6.7 pixels/µm), while the relative amount of each pigment was shown on the vertical axis (Figs 2 and 3). The results showed that there was little change in the relative amounts of chlorophyll *a* and *b* during light irradiation (Fig. 2B–E).

The relative amount of astaxanthin peaked at the cell center (horizontal axis values, 50–175 pixels) at time 0 min (Fig. 3A,B). At 10 min after light irradiation, astaxanthin concentration peaked at the cell periphery (horizontal axis values, 25–50, and 175–225 pixels) (Fig. 3A,B). There was no change in the total relative intracellular amount of astaxanthin during light irradiation (Fig. 3B). However, the relative amounts of lutein and β-carotene at the cell periphery (horizontal axis values, 25–50, and 175–225 pixels) were high at time 0 min, and these pigments were distributed throughout the cell within 10 min after irradiation (Fig. 3C–F). Therefore, lutein, β-carotene, and astaxanthin showed different intracellular dynamics in response to high-intensity light (Fig. 3C–F).

**Figure 4:**
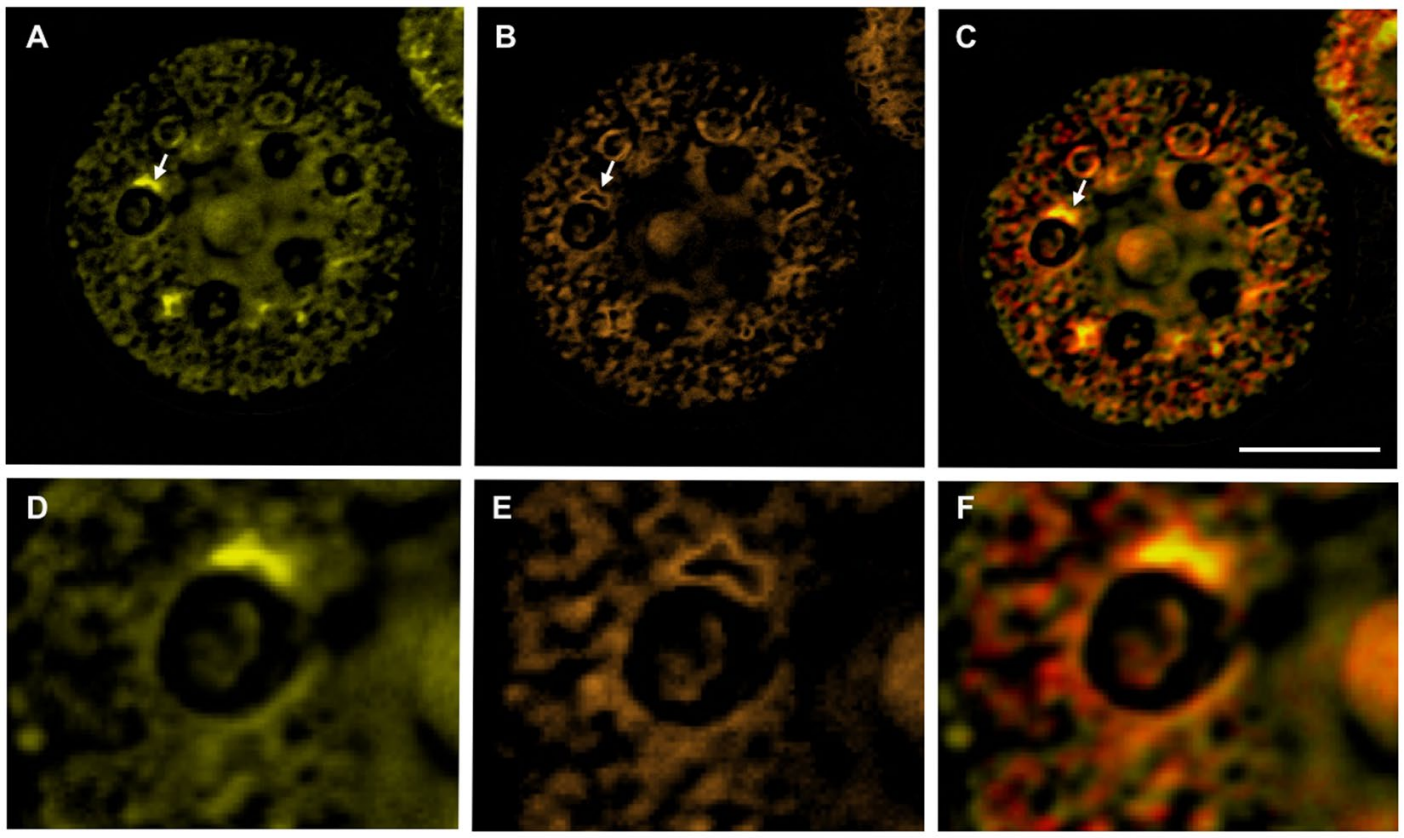
Intracellular localization of lutein and β-carotene after light irradiation. The images correspond to those obtained after 10 min in Fig. 3C,E. (**A**) Intracellular localization of lutein. (**B**) Intracellular localization of β-carotene. (**C**) Merged image of (**A**) and (**B**). (**D**), (**E**), and (**F**) are enlarged images of the regions indicated by arrows in (**A**), (**B**), and (**C**), respectively. Lutein and β-carotene are shown in yellow and orange, respectively. Scale bar, 80 pixels (=11.9 µm).

Lutein and β-carotene were nearly colocalized in the chloroplast before light irradiation (Figs 3C,E and 4). However, after light irradiation, lutein was diffusely redistributed to the nuclear periphery (Fig. 3C, time 6, 8, and 10 min; Fig. 4A), whereas β-carotene exhibited a granular distribution (Fig. 3E, time 6, 8, and 10 min; Fig. 4B). In addition, lutein accumulated around the pyrenoid, and was surrounded by β-carotene (Fig. 4C,D–F).

### Comigration of lipid droplets and astaxanthin

**Figure 5:**
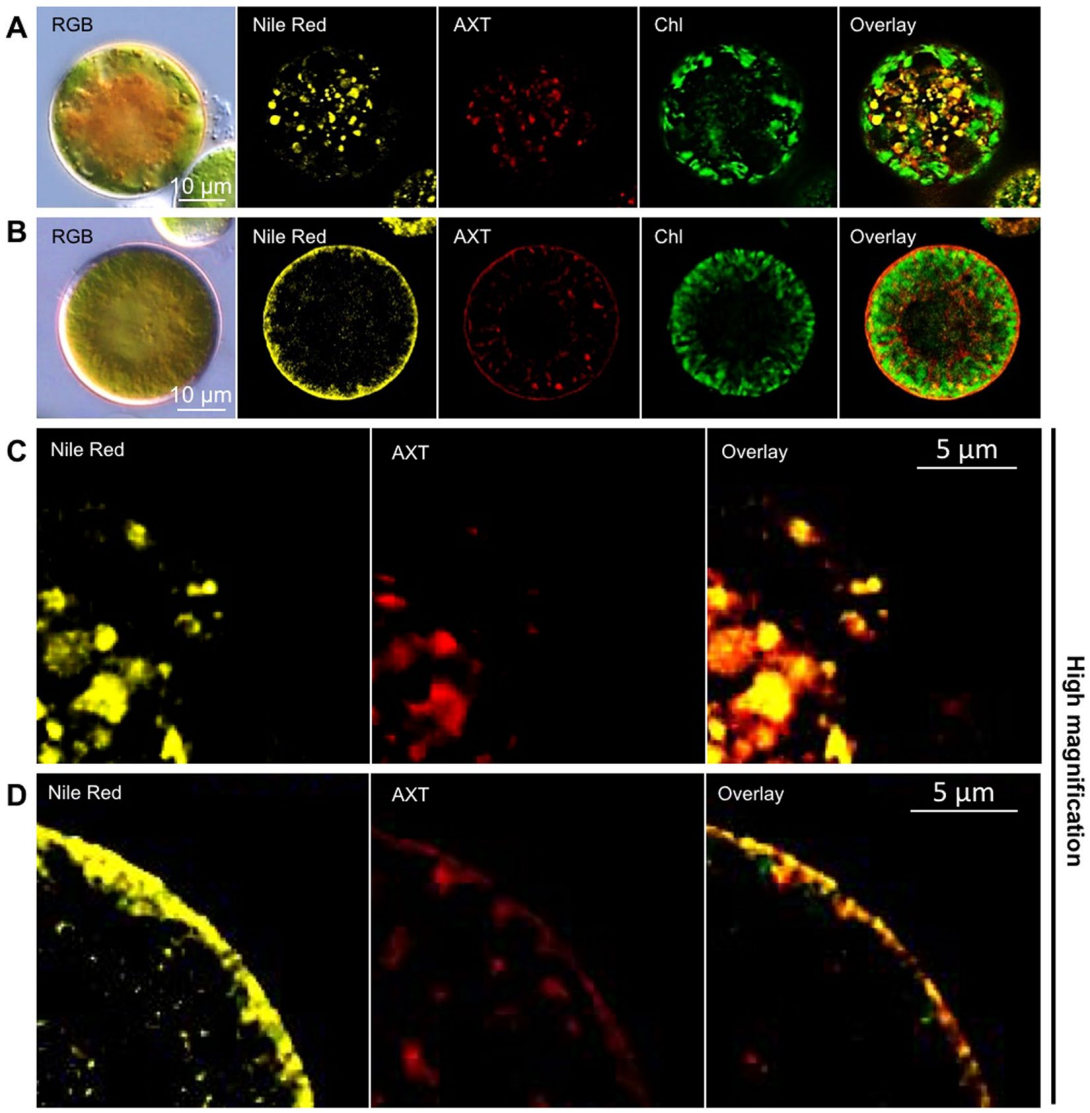
Fluorescence microscopy of lipid, astaxanthin, and chloroplasts. (**A**) Cell before light irradiation. (**B**) Cell after 10 min of light irradiation. Images are shown in the order: bright field (RGB), lipid fluorescence (Nile Red), astaxanthin autofluorescence (AXT), chloroplast autofluorescence (Chl), and overlay. (**C**) and (D) show enlargements of (**A**) and (**B**), respectively.

We investigated lipid droplet and astaxanthin dynamics using fluorescence microscopy (Fig. 5). Nile Red, a fluorescent dye that stains neutral lipids, was used to stain lipid droplets. Before light irradiation, astaxanthin was densely localized in the central part of the cell (Fig. 5A,C), as shown in the bright-field images (Fig. 5A). In the fluorescence images, both lipids and astaxanthin were mostly co-localized at the cell center before irradiation (Fig. 5C). Remarkably, some granules did not overlap in the cytoplasm. The localization of chloroplast autofluorescence differed markedly from that of lipid and astaxanthin (Fig. 5A,C). However, after light irradiation, astaxanthin was distributed diffusely throughout the cell within 10 min (Fig. 5B,D). In the fluorescence images, lipid and astaxanthin accumulated at the cell surface (Fig. 5D). These observations suggest that lipid and astaxanthin comigrate in response to light.

**Figure 6:**
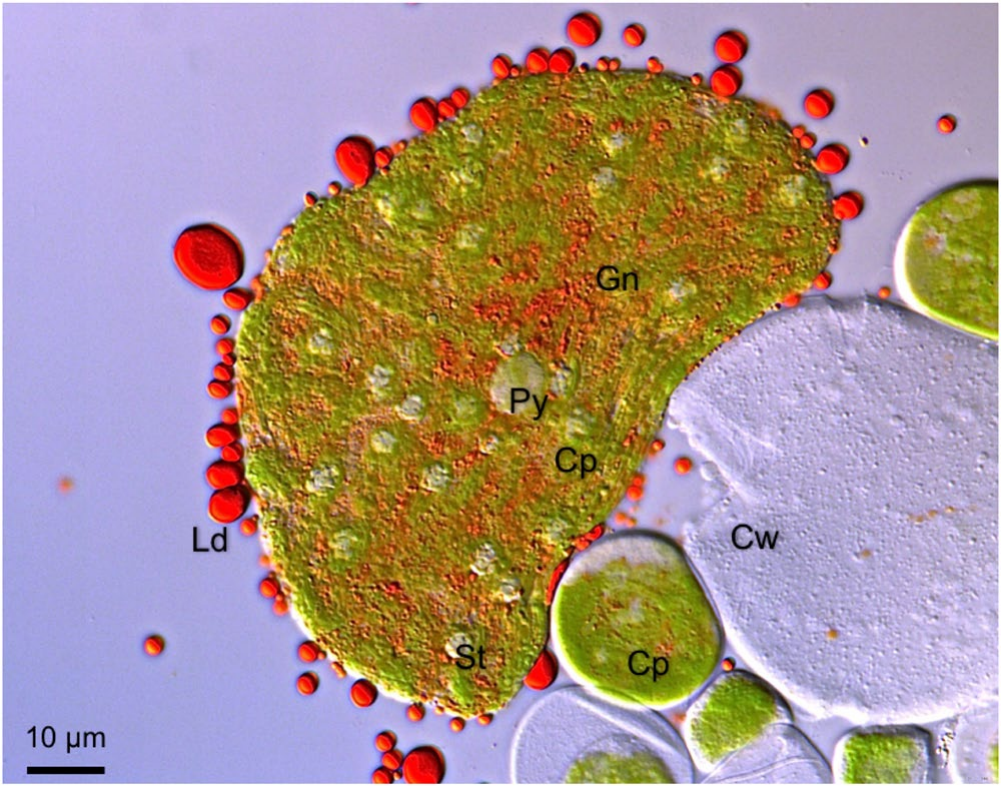
A large, spherical, cytoplasm-encapsulating chloroplast popped out from the green-red cyst and inside was a network pattern of gaps. Large and small red lipid droplets were observed around the chloroplast, and small red lipid droplets passing through a network pattern of gaps of the folded chloroplast resembled the Mars canal. Cp, chloroplast; Cw, cell wall; Gn, gap network of the chloroplast; Ld, lipid droplet; St, starch granule; Py, pyrenoid.

Green-red cysts in culture transferred to a conical flask containing 100 mL TAP medium and incubated for 1 week were not homologous in size. A 10 µL aliquot of cell suspension sandwiched between a cover glass and a microscope slide frequently contained large-diameter green-red cysts. We narrowed the clearance between the cover glass and the microscope slide by pushing them with the thumb to open a larger green-red cyst and pop out a large, spherical, cytoplasm-encapsulating chloroplast from the cell (Fig. 6). The chloroplast itself did not break because the small cysts acted as a cushion. We observed red lipid droplets around the large, hollow sphere as the immovable chloroplast popped out. Large and small red lipid droplets oozed out from the chloroplast, and a characteristic red pattern similar to the Mars canal was seen on the chloroplast. This observation supports our idea that astaxanthin freely migrates as a free lipid droplet from the center to the periphery of the cell through a large, spherical, cytoplasm-encapsulating chloroplast.

### TEM and rapid-freezing/FF-TEM of lipid droplets and chloroplasts

We fixed *Haematococcus* during encystment with KMnO_4_ to observe the red lipid droplet canal in the chloroplast by TEM (Fig. S1). Green cyst cells exhibited better-developed thylakoid membranes than green-red cyst cells. Moreover, several gaps through which lipid droplets could pass when moving from the center to the periphery of the cell were observed in gaps of the folded chloroplast. They appeared to be discrete chloroplast, but they were only cut surfaces of the folded folds of the chloroplast.

**Figure 7:**
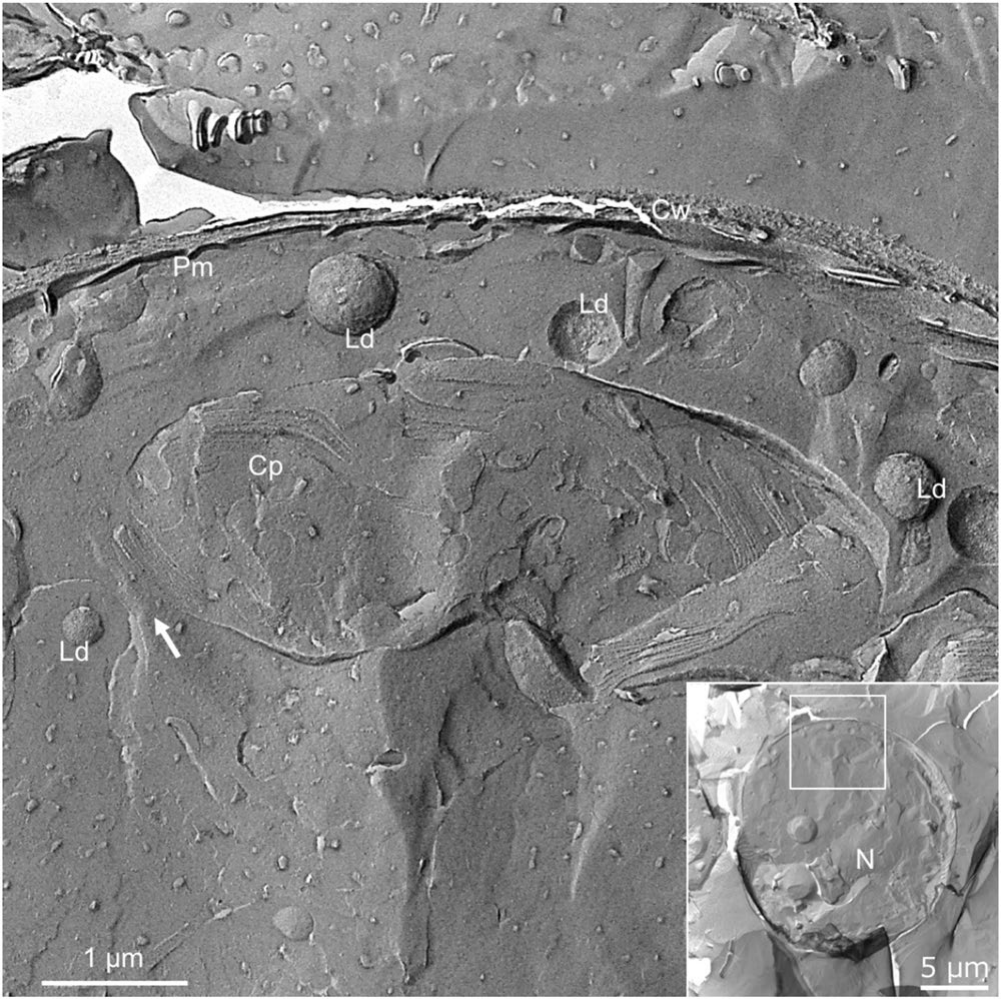
Rapid-freezing/FF-TEM image of a cell after light irradiation. The inset shows the whole cell; the area delineated by the white box is enlarged. Arrows indicate the estimated path of the chloroplast along which lipid droplets move. N, nucleus; Cp, chloroplast; Cw, cell wall; Ld, lipid droplet; Pm, plasma membrane.

KMnO_4_ does not fix lipids. Therefore, we performed rapid-freezing/FF-TEM. Cells were exposed to high-intensity light for 10 min and rapidly frozen and cleaved, and a freeze-fracture replica was prepared and observed by TEM. In green-red cyst cells before light irradiation, no lipid droplet was present near the cell wall (Fig. S2). In contrast, small lipid droplets on the surface were observed at the nucleus periphery (Fig. S2). Lipid droplets were detected near the entrance of the gap between the folded folds of the chloroplast just after light irradiation (Fig. S3). In other cells after light irradiation, several lipid droplets were lined up along the cell wall (Fig. 7). Many lipid droplets gathered in layers between the cell wall and the outside of the chloroplast, and some fused to become larger (C in Fig. S4).

Before light irradiation there were 34 and 4 lipid droplets per 50 μm^2^ at the nucleus periphery and near the cell wall, respectively. In contrast, the number of lipid droplets increased to 19 per 50 μm^2^ after light irradiation near the cell wall. In the vicinity of the chloroplast, 11 lipid droplets per 50 μm^2^ were present both before and after light irradiation (Table S1). Lipid droplets at the nucleus periphery, chloroplast region, and near the cell wall were generally 0.23–0.35 µm in diameter before light irradiation, whereas the diameter was slightly increased after light irradiation, 0.42 and 0.51 µm in the chloroplast region and near the cell wall, respectively (Table S2).

## Discussion

In this study, we applied time-lapse live-imaging and hyperspectral imaging to quantitatively analyze the intracellular diffusion of carotenoids. In the hyperspectral imaging, we focused on lutein and β-carotene, as well as on astaxanthin and chlorophylls. β-Carotene is a major carotenoid as the precursor of astaxanthin. Lutein, one of the α-carotene derivatives, is also the major carotenoid synthesized via a different metabolic pathway than astaxanthin^21^. The hyperspectral imaging of the three major carotenoids may address the fundamental issue of carotenoid dynamics during the light response in *Haematococcus*. When green-red cyst cells were exposed to high-intensity light, astaxanthin-containing lipid droplets moved rapidly toward the cell periphery (Fig. 1). In contrast, when the cells were returned to dark conditions, astaxanthin returned to the cell center (Fig. 1). This is likely because the light-shielding effect of astaxanthin is too strong under normal light conditions (<45 µmol photons m^−2^ s^−1^), such that the astaxanthin-containing lipid droplets could be reversed to the nucleus periphery to expose the chloroplast and to absorb light efficiently under normal light conditions. However, a shield of astaxanthin remained at the cell periphery for a short period under dark conditions. It seems that different diffusion mechanisms are at work in the central and centrifugal directions.

In plants and algae, β-carotene, a precursor of astaxanthin, is synthesized from lycopene by lycopene β-cyclase (LCY-b)^34,35^. In *Haematococcus*, astaxanthin accumulation starts at the nucleus periphery^36^. Nonlinear optical microscopy showed that astaxanthin accumulates in the central region of *Haematococcus* cells under stress conditions^37^. Recently, Chen *et al*. proposed that β-carotene is exported from the chloroplast to the ER, where it is converted into astaxanthin^13^. However, no studies have observed the subcellular dynamics of each carotenoid species in *Haematococcus*. The present study intended to confirm the results of molecular and physiological research using hyperspectral imaging analysis.

Recently, two mechanisms for the migration of oil globules containing astaxanthin were proposed^33^: (i) reorganization of the chloroplast and (ii) close alignment of oil globules with the ER membrane. The authors proposed that light-driven astaxanthin oil globule migration is powered by reversible associations between actin filaments and the ER–globule network. However, there was no evidence of reorganization of the chloroplast under high-intensity light. The hyperspectral analysis showed that the relative amounts and localization of chlorophyll *a* and *b* changed little during astaxanthin migration (Fig. 2B–E). This suggests that there was no movement in the large, spherical, cytoplasm-encapsulating chloroplast. However, turgor pressure explains oil globule movement. In a recent study, lipid droplets microinjected into hyphae moved by mass flow toward a lower turgor area in the oomycete *Achlya bisexualis*^38^. Because we did not measure turgor pressure gradients in this study, further research is required to determine the driving force behind the lipid droplets.

Only carotenoids responded to high-intensity light, and the photoresponsive dynamics differed between astaxanthin and the other carotenoid species. The finding that the total relative intracellular concentration of astaxanthin did not change within 10 minutes of light irradiation suggests that astaxanthin is not *de novo* synthesized at this time. Additionally, after astaxanthin migration, β-carotene and lutein localization changed from the cell margin to the whole cell (Fig. 3). According to the previous study^13^, this is temporary migration during the process of β-carotene transfer from the chloroplast to the ER. Our hyperspectral imaging results appear to support their hypothesis (Fig. S5). Another carotenoid, lutein, accumulated around the pyrenoids and was surrounded by β-carotene (Fig. 4C–F), which suggests that lutein and β-carotene are synthesized *de novo* around the pyrenoids in the protoplast, but confirmation is required.

Regarding the relationship between astaxanthin and lipid localization, lipid droplet and astaxanthin fluorescence were present at the cell center and exhibited a granular distribution before light irradiation. Following light irradiation, lipid droplets were observed at the cell periphery, and most colocalized with astaxanthin. Previous studies suggested that TAG is a prerequisite for the initiation of fatty acid–astaxanthin-ester accumulation in lipid droplets^39^. Because astaxanthin is lipid-soluble^40^ and astaxanthin accumulates around the nucleus^36^, this finding suggests that the intracellular movement of astaxanthin is equivalent to the movement of lipid droplets, as shown in Fig. 5. The lipid droplets that appeared around a large, spherical, cytoplasm-encapsulating chloroplast had a reddish color, due to astaxanthin, as shown in Fig. 6.

Wayama *et al*. used more than 350 serial sections to reconstruct 3D-TEM images during encystment to visualize subcellular relationships and changes in *Haematococcus*^6^. They fixed green coccoid cells with glutaraldehyde and KMnO_4_ to observe gaps of the chloroplast on ultrathin sections. KMnO_4_ fixation enabled clear visualization of membrane structures such as envelopes and thylakoids. In our work, TEM observations of KMnO_4_-fixed green-red cyst cells also revealed that the chloroplast has a folded structure that surrounds gaps running from the center to the periphery of the cell (Fig. S1). Although lipid droplets are uniformly covered with 6 to 9 nm particles called globule proteins^22,23^, KMnO_4_ fixation does not successfully fix lipid droplets in *Haematococcus*^6^. In our studies using rapid-freezing/FF-TEM, we observed lipid droplets and several gaps between folded folds of one chloroplast on the FF surfaces. These lipid droplets were able to pass through the gaps of the folded chloroplast from the center to the periphery of the cell. The network of the gaps of the folded chloroplast through which the red lipid droplets containing astaxanthin passed one after another looked like the canals on Mars when we looked at the entire chloroplast from a bird’s-eye view, as shown in Fig. 6. Using rapid-freezing/FF-TEM, we could not confirm the ER structure within the chloroplast gap. Although the ER–lipid droplet network model seems more likely^33^, verification of the lipid droplet movement mechanism is necessary, including turgor pressure and other membrane-bound motor proteins.

In summary, we visualized the distribution of carotenoids *in vivo* during the migration of astaxanthin. The present hyperspectral analysis revealed that subcellular dynamics in photoresponse differed between astaxanthin and other pigments, such as chlorophylls, lutein, and β-carotene. Only astaxanthin migrated through the cytoplasmic region as free lipid droplets (Fig. S5). Previous conventional red-green-blue (RGB) imaging investigations of the abundance of carotenoids were hampered by their overlapping spectral characteristics. Our results demonstrate the capacity of the hyperspectral approach to evaluate the subcellular distribution and dynamics of carotenoids with high spatial and spectral resolution. This approach is expected to be applicable to a wide range of label-free *in vivo* imaging.

## Methods

### Strain and growth conditions

*Haematococcus pluvialis* strain K-0084 (*H*. *pluvialis* was recently synonymized with *H*. *lacsutris*^5^) was obtained from the Scandinavian Culture Collection of Algae and Protozoa (SCCAP) at the University of Copenhagen. Cells were precultured at 23 °C under light at 10 µmol photons m^−2^ s^−1^ with a 12-h:12-h light (L):dark (D) cycle in a test tube (Test 30NP, Iwaki, Japan) containing 30 mL of Tris-acetate-phosphate (TAP) medium^41^, without agitation or aeration. One-month-old pre-culture (1–2 mL) was transferred to a conical flask containing 100 mL of TAP medium and incubated for 1 week at 23 °C under light at 30 ± 10 µmol photons m^−2^ s^−1^ with a 12-h:12-h L:D cycle and agitation using a magnetic stirrer (Spinpad, Frontlab, Japan) at 50–70 rpm.

### Light microscopy and time-lapse imaging

A 10-µL aliquot of cell suspension was sandwiched between two cover glasses (Matsunami No. 1, 24 × 60 mm and 18 × 18 mm), and subjected to time-lapse observation under a 100× objective lens on an inverted microscope (Eclipse Ti, Nikon, Japan). Images were captured using a color charge-coupled device (CCD) camera (DS-Fi 2, Nikon, Japan) at 15-s intervals for 10 min. A microscope light source was used (12 V/100 W halogen lamp) to provide light irradiation. To ensure reproducibility, the dimming level of the transmission illumination lamp was set at 12 V/100 W and an NCB 11 filter was placed in the optical path. For colorimetric analysis, digital images were imported into ImageJ, decomposed into the L*a*b* color space using KBI ImageJ Plugins (https://lpixel.net/en/), and red hue values were analyzed using the a* colorimetric system. ROIs were selected in the center, around the chloroplast, and immediately beneath the cell wall of green-red cyst cells, and red hue values (a*) were plotted.

### Hyperspectral imaging

Cell suspensions (10 µL) were spotted on 22 × 22 mm coverslips and covered with a glass slide. Bright-field hyperspectral images of green-red cyst cells were obtained using an AxioPlan II microscope (Zeiss, Germany) with a liquid-crystal tunable filter (VariSpec VIS, Perkin Elmer, USA) and an electron-multiplying (EM) CCD camera (ImagEM C9100-13, Hamamatsu Photonics, Japan) with 50-ms exposure (image acquisition rate per data cube, <9 fps). A (*x*, *y*, *λ*) data cube was of 512 × 512 pixels (6.7 pixels/µm) with 16-bit (2^16^) grayscale levels. The spectral resolution was set to 2 nm at wavelengths of 420–720 nm. For time-course observation, six time-lapse data cubes were obtained using the EM-CCD camera at 2-min intervals after irradiation. The pixel intensity in the spectral images was transformed to the absorbance, as described previously^32^. Multiple linear regression analysis was used to estimate the concentrations of astaxanthin, β-carotene, lutein, and chlorophyll *a* and *b* using nonnegative least squares (nnls)^42^ in R (http://www.R-project.org/). The absorbance spectra of pure pigments were used as references^32^. The relative amounts of pigments in each pixel were calculated by estimating *β* by nnls analysis:

$${\rm{Pixel}}\,{\rm{spectrum}}={\beta }_{0}+{\beta }_{1}{\rm{Ast}}+{\beta }_{2}{\rm{Car}}+{\beta }_{3}{\rm{Lut}}+{\beta }_{4}{\rm{Chla}}+{\beta }_{4}{\rm{Chlb}}+\varepsilon $$

where pixel spectrum is the observed value; Ast, Car, Lut, Chla, and Chlb are the reference absorbance spectra of astaxanthin, β-carotene, lutein, and chlorophyll *a* and *b* at each wavelength from 420 to 720 nm; and *ε* is error.

### Light and fluorescence microscopy

To visualize lipid droplets and astaxanthin, 1 µL of Nile Red (10 mM in dimethyl sulfoxide [DMSO], Polysciences, Warrington, PA, USA) was added to 9 µL of cell suspension and mixed by pipetting for 30 s. The cells were observed using a Leica DM6000B fluorescence microscope (Leica Microsystems, Germany) equipped with differential interference contrast (DIC) optics. A previous study showed that astaxanthin content was correlated with emissions at ~675 nm^43^. Nile Red signal and astaxanthin and chlorophyll autofluorescence were detected using an L5 filter cube (excitation filter, 480/40-nm band pass; suppression filter, 527/30-nm band pass), an N3 filter cube (excitation filter, 546/12-nm band pass; suppression filter, 600/40-nm band pass), and a Y5 filter cube (excitation filter, 620/60-nm band pass; suppression filter, 700/75-nm band pass), respectively. Grayscale and RGB images were obtained using a DFC360FX CCD camera (Leica Microsystems) and a DFC450C color CCD camera (Leica Microsystems), respectively.

### TEM

TEM samples were subjected to glutaraldehyde pre-fixation and KMnO_4_ post-fixation. Fixation, dehydration, and resin-embedding were performed as described by Wayama *et al*.^6^. Ultra-thin sections were observed at 100 kV with an H-7650 TEM (Hitachi High Technologies, Tokyo, Japan).

### Rapid-freezing/FF

FF was performed as described by Sekida *et al*.^44^. Green-red cyst cells were irradiated under an LED light bulb (LDA5N-E17, Toshiba, Japan) for 10 min, and then removed from the surface of the membrane filter using a razor. The cells were mounted on the holes of 3-mm single-replica gold supports, immediately frozen in nitrogen slush, and stored in liquid nitrogen. FF and metal shadowing were performed using a BalTec BAF 060 instrument (BAL-TEC, Fürstentum, Liechtenstein) at −106 °C and 1 × 10^−6^ mbar. Specimens were shadowed unidirectionally at 60° with platinum-carbon and then coated with carbon. Replicas were cleaned in a mixture of 2.5% sodium dichromate and 50% sulfuric acid for 1–2 weeks, washed with distilled water and mounted on Formvar-coated copper grids. Replicas were observed at 100 kV using an H-7650 TEM (Hitachi High Technologies).

## Electronic supplementary material

Movie S1

Movie S2

Supplementary Information
