# Supplementary material for: Carotenoid dynamics and lipid droplet containing astaxanthin in response to light in the green alga Haematococcus pluvialis
Source: Sci Rep. 2018 Apr 4;8:5617. doi: 10.1038/s41598-018-23854-w (PMC5884812; doi:10.1038/s41598-018-23854-w)
Supplement: Supplementary file 3 — Supplementary Information [file 41598_2018_23854_MOESM3_ESM.pdf]

Supplementary Information

Carotenoid dynamics and lipid droplet containing astaxanthin in response to light in the green alga *Haematococcus pluvialis*

Shuhei Ota[1,4,6]*, Aya Morita[1,6], Shinsuke Ohnuki[1], Aiko Hirata[1,2], Satoko Sekida[3], Kazuo Okuda[3], Yoshikazu Ohya[1], Shigeyuki Kawano[1,5]*

[1]Department of Integrated Biosciences, Graduate School of Frontier Sciences, The University of Tokyo, Kashiwanoha, Kashiwa, Chiba 277-8562, Japan.
[2]Bioimaging Center, Graduate School of Frontier Science, The University of Tokyo, Kashiwanoha, Kashiwa, Chiba 277-8562, Japan.
[3]Graduate School of Kuroshio Science, Kochi University, Akebono-cho, Kochi 780-8520, Japan.
[4]Present address: Center for Environmental Biology and Ecosystem Studies, National Institute for Environmental Studies, Tsukuba, Ibaraki 305-8506, Japan.
[5]Present address: Future Center Initiative, The University of Tokyo, Wakashiba, Kashiwa, Chiba 277-0871 Japan.
[6]These authors contributed equally to this work.
*Corresponding authors

Table S1 | Number of lipid droplets pre- and post-irradiation.

Number of lipid droplets (per 25 μm²)	Nucleus periphery	Chloroplast region	Near cell wall	Total
Pre-irradiation	34	11	4	49
Post-irradiation	18	11	19	48

Table S2 | Diameter of lipid droplets pre- and post-irradiation.

Diameter of lipid droplet (μm)	Nucleus periphery	Chloroplast region	Near cell wall
Pre-irradiation	0.26 (± 0.14)	0.23 (± 0.09)	0.35 (± 0.16)
Post-irradiation	0.26 (± 0.04)	0.42 (± 0.16)	0.51 (± 0.18)

* Observations were made twice independently. The mean (± standard deviation) of each observation is shown.

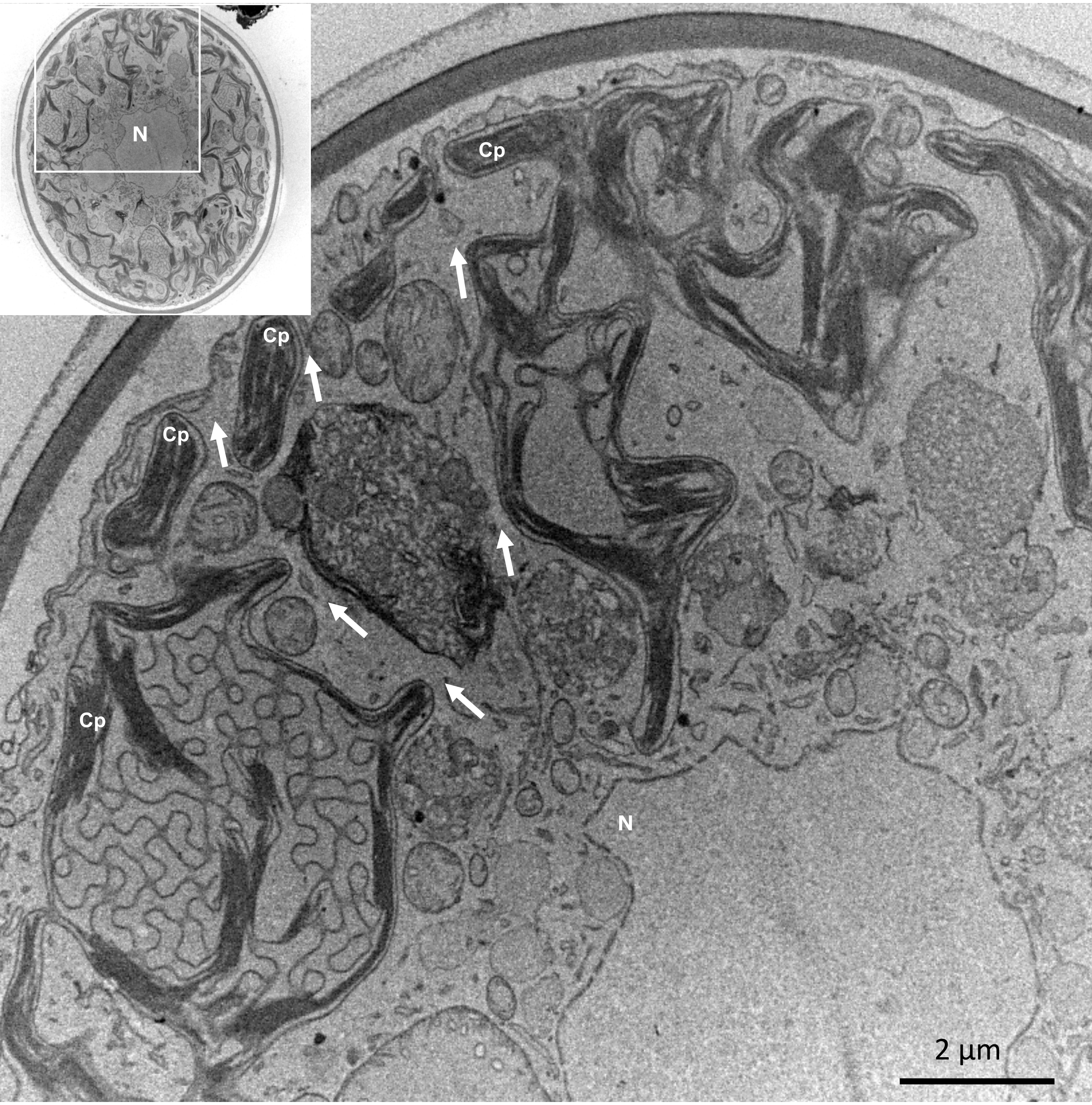

Figure S1 | TEM image of green cyst cells fixed with $KMnO_4$.
Green cells containing little astaxanthin were observed by TEM after $KMnO_4$ fixation. The inset shows the whole cell; the portion indicated by the white box is enlarged. N, nucleus; Cp: chloroplast. Arrows indicate a cytoplasmic passage in the chloroplast.

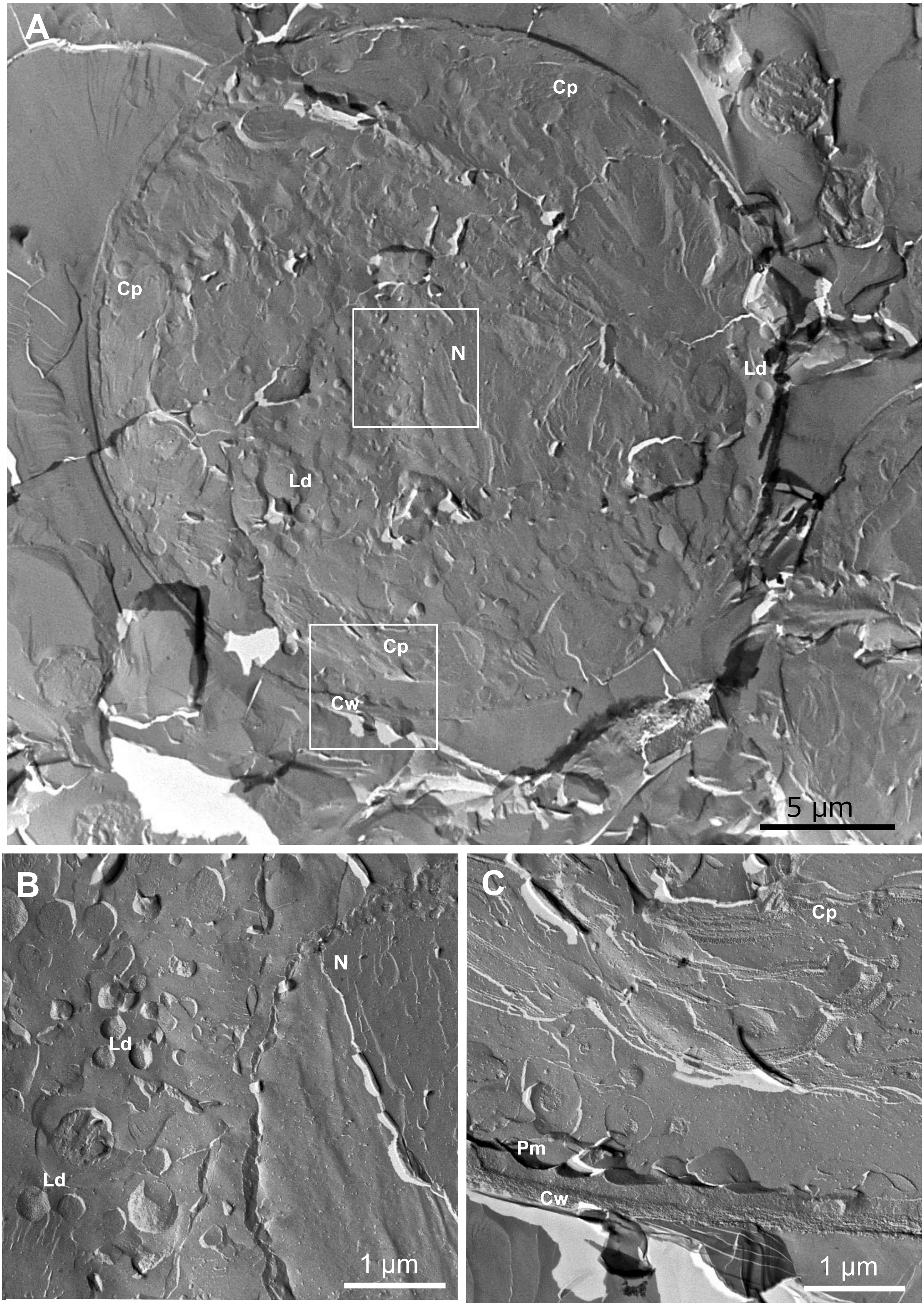

Figure S2 | A cell before light irradiation revealed by rapid-freezing/FF-TEM.
(A) Image of an FF whole cell showing general structural features. (B) Enlarged image of the region near the nucleus (white box in A). (C) Enlarged image of the region near the cell wall (white box in A). N, nucleus; Cp, chloroplast; Cw, cell wall; Ld, lipid droplet; Pm, plasma membrane.

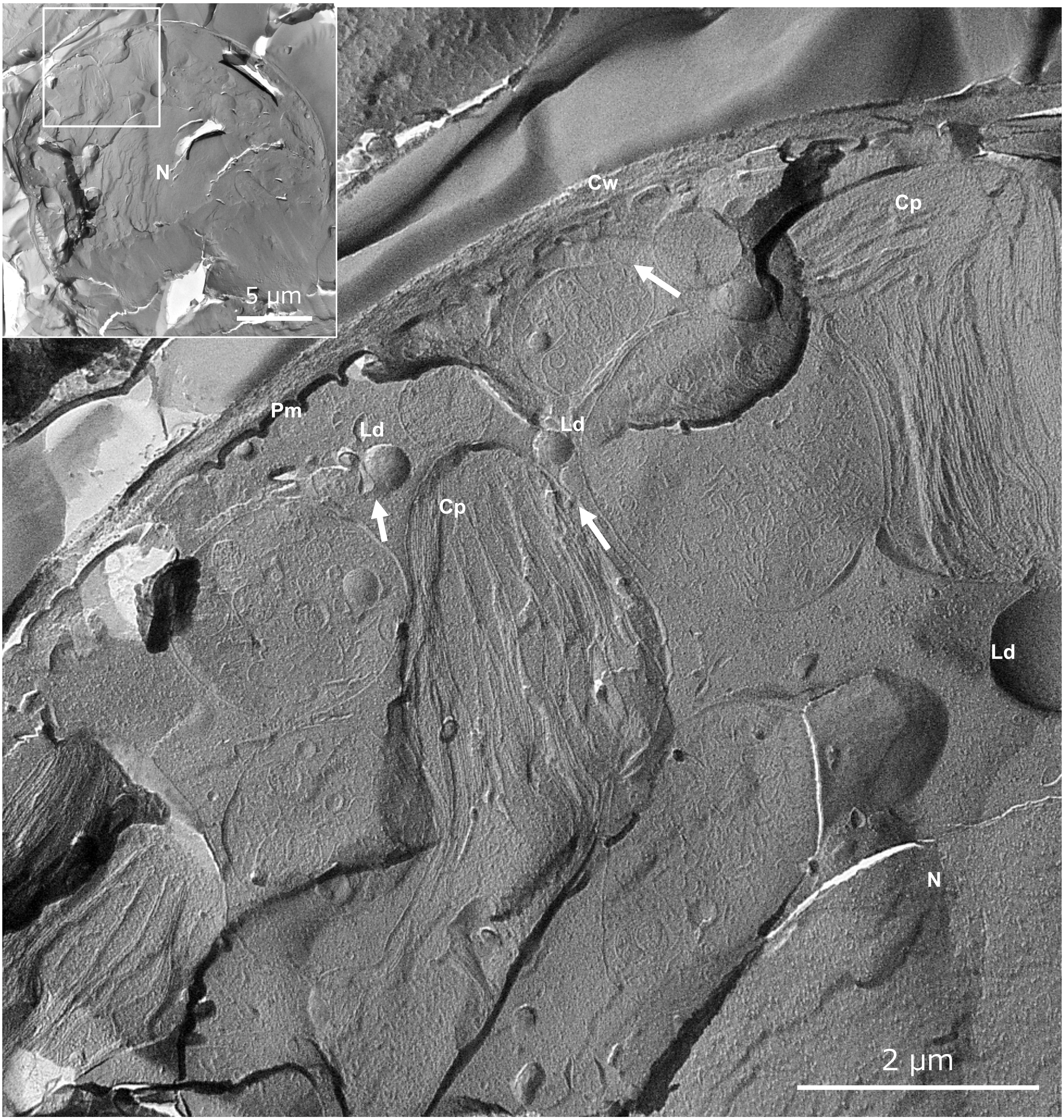

Figure S3 | Lipid droplets and gaps between folded folds of one chloroplast revealed by rapid-freezing/FF-TEM.
The inset shows the whole cell; the area delineated by the white box is enlarged. Arrows indicate the estimated gaps between folded folds of one chloroplast along which lipid droplets move. N, nucleus; Cp, chloroplast; Cw, cell wall; Ld, lipid droplet; Pm, plasma membrane.

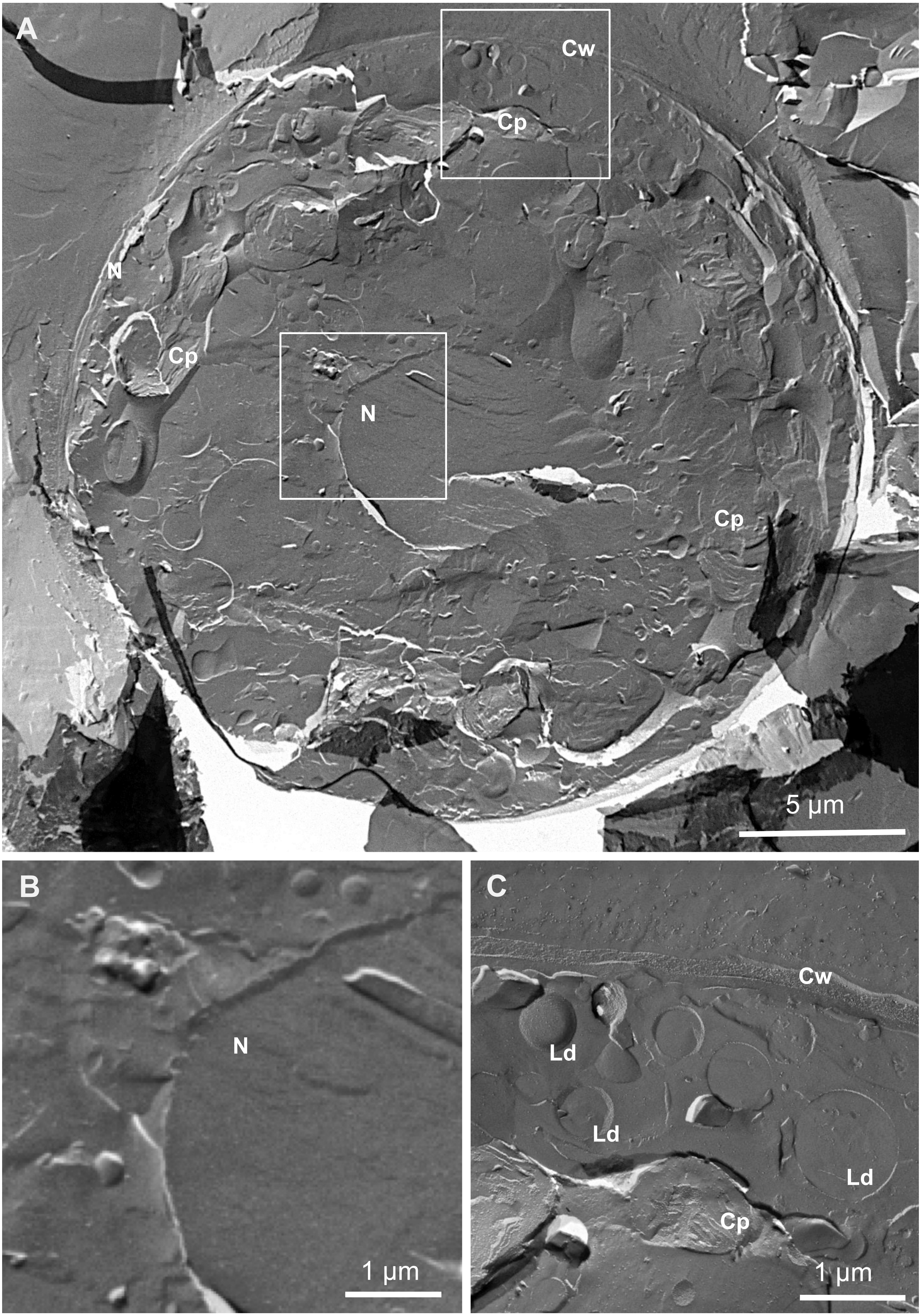

Figure S4 | Lipid droplets in layers and a fused large lipid droplet between the cell wall and the chloroplast revealed by rapid-freezing/FF-TEM.
(A) Image of an FF whole cell showing general structural features. (B) Enlarged image of the region near the nucleus (white box in A). (C) Enlarged image of the region near the cell wall (white box in A). N, nucleus; Cp, chloroplast; Cw, cell wall; Ld, lipid droplet.

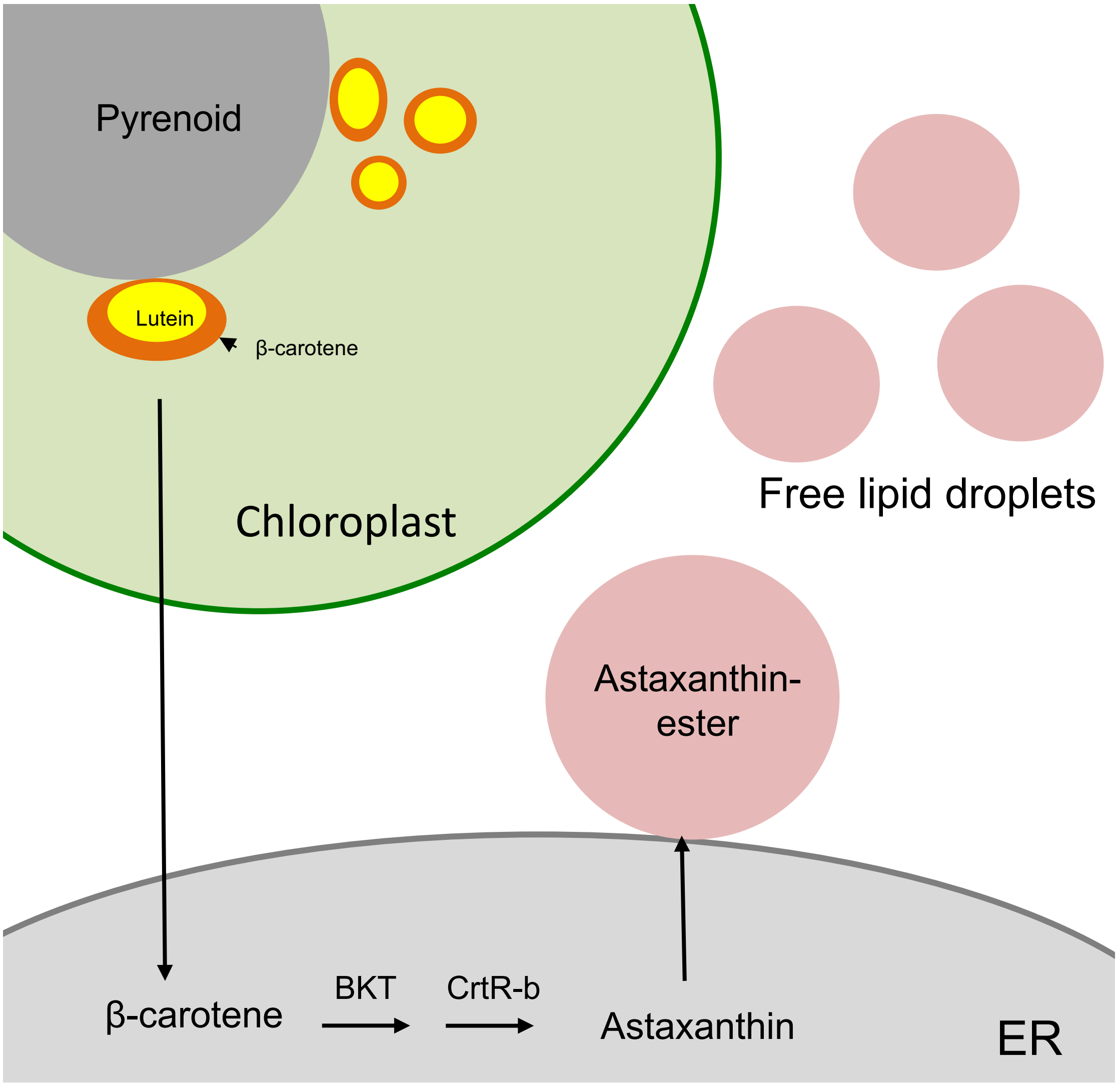

Figure S5 | Schematic illustration of astaxanthin biosynthesis in *Haematococcus*. This diagram is based on our data and the model proposed by Chen et al. (2015). Our hyperspectral imaging data demonstrate that β-carotene and lutein migrate from the chloroplast. Triacylglycerol synthesis is omitted from this diagram.
